# Dietary Blueberry and Soluble Fiber Improve Serum Antioxidant and Adipokine Biomarkers and Lipid Peroxidation in Pregnant Women with Obesity and at Risk for Gestational Diabetes

**DOI:** 10.3390/antiox10081318

**Published:** 2021-08-22

**Authors:** Arpita Basu, Jeannette Crew, Jeffrey L. Ebersole, Jefferson W. Kinney, Arnold M. Salazar, Petar Planinic, James M. Alexander

**Affiliations:** 1 Department of Kinesiology and Nutrition Sciences, University of Nevada at Las Vegas, Las Vegas, NV 89154, USA; crewj1@unlv.nevada.edu; 2 School of Dental Medicine, University of Nevada at Las Vegas, Las Vegas, NV 89106, USA; jeffrey.ebersole@unlv.edu; 3 Department of Brain Health, University of Nevada at Las Vegas, Las Vegas, NV 89154, USA; Jefferson.Kinney@unlv.edu (J.W.K.); arnold.salazar@unlv.edu (A.M.S.); 4 Department of Obstetrics & Gynecology, Kirk Kerkorian School of Medicine, University of Nevada at Las Vegas, Las Vegas, NV 89102, USA; pplaninic@gmail.com (P.P.); james.alexander@unlv.edu (J.M.A.); 5 Valley Health System, Las Vegas, NV 89119, USA

**Keywords:** pregnancy, obesity, glutathione, blueberries, adipokines, malondialdehyde

## Abstract

Pregnancies affected by obesity are at high risk for developing metabolic complications with oxidative stress and adipocyte dysfunction contributing to the underlying pathologies. Few studies have examined the role of dietary interventions, especially those involving antioxidants including polyphenolic flavonoids found in fruits and vegetables on these pathologies in high-risk pregnant women. We conducted an 18 gestation-week randomized controlled trial to examine the effects of a dietary intervention comprising of whole blueberries and soluble fiber vs. control (standard prenatal care) on biomarkers of oxidative stress/antioxidant status and adipocyte and hormonal functions in pregnant women with obesity (*n* = 34). Serum samples were collected at baseline (<20 gestation weeks) and at the end of the study period (32–26 gestation weeks). Study findings showed maternal serum glutathione and antioxidant capacity to be significantly increased, and malondialdehyde to be decreased in the dietary intervention vs. control group (all *p* < 0.05). Among the adipokine biomarkers, serum plasminogen activator inhibitor-1 and visfatin, as biomarkers of adipocyte dysfunction and insulin resistance, were also decreased following dietary intervention (all *p* < 0.05). These findings support the need for supplementing maternal diets with berries and fiber to improve oxidative stress and risks of metabolic complications during pregnancy.

## 1. Introduction

Maternal obesity has been shown to increase pregnancy complications and increase the risk of gestational diabetes mellitus (GDM) with its well-studied complications including increased risk of fetal macrosomia, shoulder dystocia with injury to the delivering fetus, and neonatal hypoglycemia [1,2]. Diet and lifestyle factors are the cornerstone of weight management in pregnancy and a decrease in excess gestational weight gain has been demonstrated to reduce pregnancy complications and lowers the risk of GDM [3]. Our previous study has focused on clinical endpoints including overall weight gain during pregnancy and clinical outcomes as well as impact on maternal glucose and lipid values [4,5]. However, little attention has been given to the impact of dietary interventions on biomarkers of antioxidant and endocrine status in pregnancy.

Oxidative stress and antioxidant status play an important role in normal pregnancy, as well as in complications, such as GDM and abortions [6]. Normal pregnancy is associated with an increase in oxidative stress as well as an increased synthesis and activities of antioxidant enzymes to facilitate the placental and fetal development and related metabolic functions [7,8]. An imbalance in antioxidant status and lipid peroxidation has been reported in pregnancies complicated by GDM vs. healthy pregnant women [9,10]. In these studies, lipid peroxidation measured as malondialdehyde (MDA) was elevated, and antioxidant cofactors, such as glutathione or selenium was reduced, while antioxidant enzymes were mostly non-different or increased to counteract elevated oxidative stress. Again, most of these studies were conducted in women with pregnancy complications, and thus, little is known about how maternal obesity, and dietary antioxidants affect oxidative stress and antioxidant biomarkers in early vs. late pregnancies. In a case–control study of obese and lean pregnant women (*n* = 15/group), a high ratio of oxidized and reduced glutathione was reported in obese mothers vs. lean controls at mid-gestation [11]. In another cross-sectional study of women with pre-gestational normal weight or obesity, placental expression of the glutathione peroxidase activity was lower in obese vs. normal weight women [12]. In addition, increasing adiposity can also lead to perturbations in metabolism via secretion of adipokines and hormones, such as leptin, plasminogen activator inhibitor-1 (PAI-1), resistin, and visfatin, which have been associated with increased risks of GDM and related pregnancy complications [13,14,15]. These biomarkers, reflecting maternal oxidative stress/antioxidant status and adiposity and metabolic functions must be evaluated during high-risk pregnancies. However, there is a scarcity of data on the role of diet and lifestyle interventions on these biomarkers during pregnancy, and this leads to the scope of the current study. We selected a panel of antioxidant enzymes/oxidative stress markers, cofactors and adipokines based on their observed responses to dietary interventions involving polyphenols in clinical studies reported by our group, [16,17,18] as well as by others [19,20] in adults with obesity or the metabolic syndrome.

Among the dietary bioactive compounds that have been shown to exert antioxidant effects and reduce cardiometabolic risks, dietary berries have shown some promising effects in clinical and observational studies [21,22,23]. Our group conducted several trials and reported improved cardiometabolic risks in non-pregnant adults following dietary berry intervention, primarily as blueberries, strawberries, or cranberry juice [24,25,26]. In addition, soluble fiber which is an essential component of berries and shown to be beneficial in diabetes [27,28] is not consumed at adequate levels by pregnant women [29]. Keeping in view the lack of such studies in pregnant women with obesity and cardiometabolic risks, we performed a clinical trial to examine effects following dietary blueberry and soluble fiber supplementation vs. standard prenatal care as control. Results showed improvements in excess gestational weight gain (GWG), blood glucose, and C-reactive protein as previously reported [4]. We aimed to examine the effects of this dietary intervention on antioxidant/oxidative stress, hormonal, and adipokine biomarkers in serum samples from the same group of women.

## 2. Methods

### 2.1. Study Design and Participants

The study design has been described in detail previously [4]. Briefly, this RCT was conducted at the Department of Obstetrics and Gynecology at the UNLV School of Medicine. This was a randomized parallel arm study in which participants were randomized to one of two groups: intervention (blueberry + soluble fiber) or control (standard prenatal care) group. The study was approved by the Human Ethics Committee at UNLV (IRB#1155039). All participants provided informed consent for the study. The inclusion and exclusion criteria have been previously described [4]. Briefly, eligibility criteria involved maternal obesity (BMI ≥ 30) and gestational age < 20 weeks with high risk for GDM, singleton pregnancy, and no pre-gestational chronic conditions. Samples for this study were collected at baseline (<20 weeks gestation) and at end of the 18-week intervention (32–36 weeks gestation). Participants in the intervention group were supplemented with 2 cups of whole blueberries and 12 g of soluble fiber (partially hydrolyzed guar gum; Nutrisource^®^ Nestle Health Science) daily for 18 weeks. This dose of fiber was selected to supplement the habitual low fiber intake of our participants and meet the dietary fiber requirements of approximately 30–35 g/day [30]. The nutritional value has been previously reported with the blueberries adding approximately 160 kcal, 1600 mg of total polyphenols, and 700 mg of anthocyanins to the maternal diet [4]. Dietary intakes were recorded based on 24-h food recalls throughout the study.

### 2.2. Antioxidant Biomarkers

Antioxidant biomarkers were measured in maternal stored serum samples using procedures as follows: serum catalase was measured using Catalase-520 (Oxis Research, Portland, Oregon) spectrophotometric assay based on the manufacturer’s protocol. The average inter-assay CV was 5.6%. Glutathione peroxidase (GPX) was measured by using GPx-340 (Oxis Research) based on the manufacturer’s protocol. The average inter-assay CV was 6.3%. Glutathione reductase (GR) and superoxide dismutase (SOD) activity were measured using commercially available kits (Cayman Chemical, Ann Arbor, Michigan) in accordance with the manufacturer’s protocol. The average inter-assay CV was 4.5 and 5.3%, respectively. Reduced glutathione was measured as previously described by Beutler et al. [31] based on the absorbance of the yellow thiolate anion at 412 nm. The average inter-assay CV was 4.3%. Serum antioxidant capacity was measured using the metmyoglobin assay developed by Miller et al. [32]. The average inter-assay CV was 6.8%. Serum nitrite was measured using the Griess Reagent System (Promega Corporation, Madison, WI) with a mean inter-assay CV of 4.6%. Serum malondialdehyde (MDA) levels were measured by the method of Jain et al. [33] based on the reaction of MDA with thiobarbituric acid to produce a complex that can be determined spectrophotometrically at 532 nm.

### 2.3. Adipokine and Hormonal Biomarkers

Adipokine and hormonal biomarkers were measured in maternal serum samples using Bio-Plex Pro™ Human Diabetes Panel magnetic bead assays (Bio-Rad Laboratories, Hercules, CA, USA), in accordance with the manufacturer’s instructions with modifications as previously described [17]. The variables measured and their corresponding intra-assay CVs were C-peptide ~3%, ghrelin ~4%, gastric inhibitory polypeptide (GIP) ~6%, glucagon-like peptide-1 (GLP-1) ~4.5%, glucagon ~5%, leptin ~4%, plasminogen activator inhibitor-1 (PAI-1) ~3%, resistin ~4%, and visfatin ~5%.

### 2.4. Dietary Intake of Antioxidants

Maternal dietary intakes of micronutrient trace elements with a role in antioxidant/oxidative stress pathways and enzyme activities (iron, copper, zinc, selenium, manganese) were determined using ESHA’s Food Processor^®^ Nutrition Analysis software. Participants were asked to maintain their 24-h food recalls for three days per week at baseline and at the end of study. The antioxidant vitamins, macronutrients, and caloric intake have been previously described [4].

### 2.5. Statistical Analysis

Descriptive statistics were calculated data that have been presented as means ± standard deviations for the continuous variables. Our primary objective for the current analysis was to determine if the antioxidant and adipokine biomarkers were significantly different between the dietary intervention and control groups over the period of the 18 gestation weeks of the study. To address this primary question, we employed a mixed model ANOVA to determine the effects of time, treatment (group), and interaction. Outcomes were modeled as repeated measures, with the subject as a random effect and with unstructured variance for treatment/time. We also conducted a secondary analysis to examine if there were any significant correlations among selected antioxidant and adipokine biomarkers in a model adjusted for maternal age, BMI and prenatal vitamin use in pooled analysis of all participants. To address this secondary question, we used a Pearson correlation coefficients analysis. As previously reported, we had 80% power to detect differences with at least 12 participants per group in this trial [4]. All *p*-values were two-tailed, and main effects and interaction effects were considered if <0.05. Analyses were performed using SAS (version 9.4; SAS Institute Inc., Cary, NC, USA).

## 3. Results

The baseline characteristics of the study participants have been previously described and a total of 34 women completed the trial [4]. Briefly, women in both groups had a baseline BMI > 30, were primarily of Hispanic origin and the prevalence of prenatal vitamin use was 35% at baseline. As previously reported, we observed a significant decrease in GWG, CRP and postprandial blood glucose in the intervention vs. control group [4].

Table 1 presents the maternal serum antioxidant biomarkers at baseline and end of the study. Among the antioxidant biomarkers, maternal serum glutathione and antioxidant capacity were significantly increased at the end of dietary intervention vs. control group. In addition, MDA significantly decreased following the dietary intervention vs. control (*p* < 0.05). No statistical differences were noted in case of maternal antioxidant enzyme activities (catalase, GR, GPX, and SOD) between the two groups.

As shown in Table 2, among the maternal adipokines and hormonal biomarkers, maternal serum PAI-1 and visfatin were significantly decreased at the end of dietary intervention vs. control group (*p* < 0.05).

We also examined adjusted correlations among selected biomarkers of maternal antioxidant and adipokine biomarkers using a pooled analysis of 34 participants who completed the trial (Table 3). We observed a significant inverse correlation of serum glutathione and antioxidant capacity with PAI-1 at baseline (r: −0.32 and −0.28, respectively) and end of study (r: −0.30 and −0.33, respectively) timepoints in adjusted models. Another significant correlation was a positive association of maternal serum MDA with visfatin at both time points (baseline r: 0.38 and study end r: 0.35) in adjusted analyses (*p* < 0.05).

Finally, we examined differences in maternal dietary intakes of trace elements with key roles in cellular antioxidant activities. As presented in Table 4, maternal habitual dietary intake of selenium was significantly increased over time, while no differences were noted in dietary intakes of iron, copper, zinc, and manganese. We previously reported no differences in dietary intakes of antioxidant vitamins (C and E), fruits and vegetables in the intervention and control groups throughout the study [4].

## 4. Discussion

We observed significant changes in maternal serum antioxidant and adipokine biomarkers following an intervention of blueberries and soluble fiber when compared to standard prenatal care group for a period of 18 gestation weeks in women at high risk for developing metabolic complications. Among the antioxidant biomarkers measured, maternal serum levels of reduced glutathione and antioxidant capacity increased, while MDA, a biomarker of lipid peroxidation, decreased following the dietary intervention. Among the adipokine biomarkers, maternal serum PAI-1 as well as visfatin decreased at the end of the dietary intervention as compared to the control group. We also observed a significant inverse correlation of maternal glutathione and antioxidant capacity with PAI-1, and a positive correlation between MDA and visfatin in models adjusted for maternal covariates, such as age, BMI, and prenatal vitamin use. These findings demonstrate an association between dietary intervention with antioxidants rich berries as well as optimized weight gain and an improved oxidative stress environment. It suggests that the low cost, well tolerated intervention in our study can improve pregnancy outcomes.

Obesity itself has been associated with increased oxidative stress and reduced antioxidant status [34], and thus pregnancies affected by obesity are more vulnerable to oxidative stress than those with normal pre-gestational body weight [35]. This suggests that adequate antioxidant protection throughout pregnancies at risk for obesity-related metabolic complications can improve outcomes. Unfortunately, antioxidant vitamins, such as vitamins C and E may not effectively ameliorate oxidative stress as evidenced by several clinical trials in which antioxidant vitamin supplementation showed no effects in reducing risks of hypertensive complications of pregnancy, such as PE in high-risk women [36,37,38]. Plant-based Mediterranean (Med) or the Dietary Approaches to Stop Hypertension (DASH) diets containing a combination of several dietary bioactive compounds have been shown to be effective in reducing metabolic complications during pregnancy, such as GDM and PE in some studies, although not in others [39,40,41]. These studies do not always report maternal antioxidant, and hormonal and adipokine biomarkers, which may be important in determining their role in these metabolic complications, and may help explain the different results. When antioxidant activity has been reported, diet has been demonstrated to be effective in improving the antioxidant status. In a study of a sample size comparable to ours, Asemi et al. reported increases in maternal plasma antioxidant capacity and glutathione following DASH diet intervention for four weeks in women with GDM [39]. In another feeding trial, soy protein consumption led to improved maternal antioxidant capacity and glutathione levels in women with GDM [42]. In a third study of women at risk for PE, garlic supplementation was also shown to increase maternal plasma glutathione levels vs. placebo treatment [43]. The clinical observation that polyphenolic flavonoids, such as those found in blueberries can increase cellular glutathione synthesis and increase antioxidant capacity are consistent with these findings [44,45]. While the antioxidant enzyme activities did not differ between groups, there is a possibility that by increasing glutathione as a cofactor for the GR and GPX enzyme functions [46], the blueberry intervention prevented changes in their activities at the end of the study.

Adipokines, such as leptin, resistin, and visfatin, have been shown to be involved in signaling pathways between adipose tissue and key organs that are affected by insulin action, such as the skeletal muscle, liver, and pancreas [47,48]. Circulating levels of PAI-1 have been associated with insulin resistance and vascular complications [49,50]. As a result, maternal circulating adipokines have been correlated with risks of GDM and preeclampsia in observational studies [14,15]. Paradoxically, dietary intervention studies in pregnant women report effects on GWG but not on these biomarkers that contribute to obesity-related pathologies in pregnancy. Our study thus addresses this gap, as we measured a defined panel of maternal biomarkers of adipokines, as well as hormones that control food intake and glucose metabolism. A whole grain-based dietary intervention was shown to decrease serum PAI-1 in non-pregnant adults over a four-week period [51]. In addition, selected trials also show decreases in PAI-1 following a low carbohydrate diet, whole grain wheat, and omega-3-fatty acid supplementation in adults with cardiometabolic risks [52,53,54]. These findings, together with ours, warrant larger trials to identify adipokines that can be modulated by dietary bioactive compounds in reducing risks of metabolic complications during pregnancy in women with obesity.

Since our dietary intervention involved a combination of blueberries and soluble fiber, identifying their individual effects cannot be done accurately. While most of the effects on antioxidant biomarkers can be attributed to the high antioxidant content in blueberries [24], a few studies have reported antioxidant and anti-inflammatory effects of different kinds of soluble fiber in animal models of diabetes [55,56], and improvement of hyperglycemia in adults with diabetes [57,58]. Keeping in view the lack of studies involving soluble fiber supplementation in pregnant women, our findings warrant further investigation in larger trials.

Although weak, we also observed a positive correlation between serum lipid peroxidation and visfatin at baseline and at the end of study in our pooled sample analysis. Our findings are supported by a few observations in which maternal circulating MDA were positively correlated with adipokines in GDM [59]. Of interest, maternal serum visfatin has been shown to regulate oxidative stress and has been identified as a novel biomarker in predicting metabolic complications in obesity during pregnancy [60,61]. In line with this observation, we also found protective associations of maternal glutathione and serum antioxidant capacity with PAI-1, which may indicate that endogenous antioxidants, and subsequently an antioxidant-rich diet that promotes healthy GWG can be protective against metabolic complications in pregnancy [62,63].

The limitations of our study include the following: a sample of high-risk pregnant women with obesity and family history and/or history of GDM as a result of which findings cannot be generalized to women with normal gestational weight gain and at low risk for complications. Intervention initiated at <20 weeks gestation and biomarkers were measured at only two time points, and this precludes examining effects over the course of gestation, such as at first, second and third trimesters of gestation. Finally, we did not examine effects on these biomarkers during the pre-gestational and postpartum phases which are critical for physiological comparisons and this must be addressed in future studies. On the other hand, our study has unique strengths as follows: We measured a comprehensive panel of biomarkers involving maternal serum antioxidant activity, oxidative stress, and adipokines before and after dietary intervention of blueberries and soluble fiber vs. control group that has not been reported in other studies. We also measured maternal intakes of dietary trace elements, such as selenium, which are relevant to antioxidant enzyme activities, and found no differences between groups. Thus, the effects on antioxidant biomarkers can be attributed to the daily dietary intervention of blueberries and soluble fiber for 18 gestation weeks.

## 5. Conclusions

A healthy diet comprised of dietary bioactive compounds can ameliorate obesity-related pathologies by improving the oxidative stress and antioxidant environment seen during pregnancies in women with obesity. Our study actively identifies antioxidant and adipokine biomarkers that are responsive to blueberry and soluble fiber supplementation in pregnant women with obesity and at high risk for gestational diabetes. The increases in serum glutathione and antioxidant capacity, as well as decreases in lipid peroxidation, PAI-I and visfatin reflect improved oxidative stress, inflammation, and adiposity function in these women and are consistent with the findings of improved clinical outcomes we previously reported.

## Figures and Tables

**Table 1:** Serum antioxidants and oxidative stress biomarkers in women with obesity and at risk for gestational diabetes in dietary intervention vs. control group for a period of 18 gestation weeks.

Variable by Time	Intervention ^1^	Control ^2^	*p*-Value ^3^ (Group)
Serum catalase, U/mL			
Baseline	48 ± 13	56 ± 15	0.27
End	62 ± 14	47 ± 10	
Serum glutathione, µM *			
Baseline	880 ± 320	914 ± 370	**0.03**
End	1236 ± 612	1107 ± 532	
Serum GR, nmol/min/mL			
Baseline	65 ± 21	72 ± 21	0.44
End	54 ± 18	68 ± 19	
Serum GPX, mU/mL			
Baseline	18 ± 8	22 ± 10	0.34
End	24 ± 11	31 ± 15	
Serum SOD, U/mL			
Baseline	0.04 ± 0.03	0.05 ± 0.03	0.39
End	0.03 ± 0.02	0.04 ± 0.02	
Serum nitrite, µM			
Baseline	21 ± 12	31 ± 11	0.31
End	28 ± 15	25 ± 9	
Serum antioxidant capacity, µmol/L			
Baseline	3.6 ± 2.2	4.8 ± 3.2	**0.01**
End	6.2 ± 4.5	5.5 ± 3.5	
Serum MDA, nmol/mL *			
Baseline	4.23 ± 2.31	5.13 ± 3.21	**0.02**
End	2.45 ± 1.18	4.65 ± 2.18	

Data presented as means ± SD. N = 17/group. Abbreviations: GR = glutathione reductase; GPX = glutathione peroxidase; MDA = malondialdehyde; PAI-1 = plasminogen activator inhibitor-1; SOD = superoxide dismutase. ^1^ Blueberry + soluble fiber. ^2^ Standard prenatal care. ^3^ P from mixed model ANOVA. *p* < 0.05 in bold font. * Significant time effect; different from baseline (*p* < 0.05).

**Table 2:** Serum adipokines and hormonal markers in women with obesity and at risk for gestational diabetes in dietary intervention vs. control group for a period of 18 gestation weeks.

Variable by Time	Intervention ^1^	Control ^2^	*p*-Value ^3^ (Group)
Serum C-peptide, pg/mL			
Baseline	2801 ± 1159	3081 ± 1660	0.56
End	2422 ± 1227	2517 ± 1289	
Serum ghrelin, pg/mL			
Baseline	242 ± 187	178 ± 159	0.37
End	197 ± 206	199 ± 189	
Serum GIP, pg/mL			
Baseline	1027 ± 432	1103 ± 705	0.42
End	927 ± 469	1080 ± 632	
Serum GLP-1, pg/mL *			
Baseline	664 ± 95	558 ± 152	0.37
End	417 ± 99	448 ± 112	
Serum glucagon, pg/mL *			
Baseline	1090 ± 171	1091 ± 134	0.38
End	795 ± 88	860 ± 124	
Serum leptin, pg/mL			
Baseline	17,877 ± 6153	18,199 ± 11,599	0.42
End	14,482 ± 6184	13,881 ± 6825	
Serum PAI-1, pg/mL *			
Baseline	4407 ± 626	3834 ± 616	**0.02**
End	3329 ± 1019	3992 ± 619	
Serum resistin, pg/mL			
Baseline	8742 ± 2705	9321 ± 1845	0.37
End	8280 ± 2295	7160 ± 1902	
Serum visfatin, pg/mL			
Baseline	8862 ± 4406	8323 ± 1056	**0.03**
End	3409 ± 1002	6591 ± 5851	

Data presented as means ± SD. N = 17/group. Abbreviations: GIP = gastric inhibitory polypeptide; GLP-1 = glucagon-like peptide-1; PAI-1 = plasminogen activator inhibitor-1. ^1^ Blueberry + soluble fiber. ^2^ Standard prenatal care. ^3^ P from mixed model ANOVA. *p* < 0.05 in bold font. * Significant time effect; different from baseline (*p* < 0.05).

**Table 3:** Partial correlation coefficients among maternal serum antioxidant, lipid oxidation and adipokine biomarkers adjusted for maternal age, BMI, and prenatal vitamin use at baseline and end of study.

Variable by Time	Leptin	PAI-1	Resistin	Visfatin
Glutathione				
Baseline	0.04	**−0.32**	0.04	0.03
End	0.05	**−0.30**	0.03	0.02
Antioxidant capacity				
Baseline	0.05	**−0.28**	−0.02	0.03
End	0.06	**−0.33**	−0.02	0.02
MDA				
Baseline	0.07	0.06	0.03	**0.38**
End	0.05	0.05	0.02	**0.35**

N = 34 at baseline and end of study. Abbreviations: BMI = body mass index; GR = glutathione reductase; GPX = glutathione peroxidase; MDA = malondialdehyde; PAI-1 = plasminogen activator inhibitor-1; SOD = superoxide dismutase. *p* < 0.05 in bold font.

**Table 4:** Maternal dietary intakes of trace elements as antioxidants following dietary intervention vs. control for 18 gestation weeks.

Variable by Time	Intervention ^1^	Control ^2^	*p*-Value ^3^ (Group)
Dietary iron, mg/d			
Baseline	20 ± 3	19 ± 5	0.46
End	21 ± 3	20 ± 4	
Dietary Copper, mg/d			
Baseline	1.2 ± 0.6	0.8 ± 0.7	0.29
End	1.3 ± 0.7	1.1 ± 0.9	
Dietary zinc, mg/d			
Baseline	8 ± 7	9 ± 7	0.34
End	11 ± 5	10 ± 6	
Dietary selenium, µg/d *			
Baseline	131 ± 61	151 ± 55	0.33
End	176 ± 84	138 ± 68	
Dietary manganese, mg/d			
Baseline	4.2 ± 2.6	3.8 ± 2.7	0.42
End	5.3 ± 3.7	4.6 ± 1.9	

Data presented as means ± SD. N = 17/group. ^1^ Blueberry + soluble fiber. ^2^ Standard prenatal care. ^3^ P from mixed model ANOVA. * Significant time effect; different from baseline (*p* < 0.05).

## Data Availability

All generated data in this study are included in the article.
